# SPP1 in notochord cells modulates intervertebral disc degeneration through CD44 recognition by macrophages based on single-cell transcriptome analysis

**DOI:** 10.1097/JS9.0000000000003454

**Published:** 2025-10-07

**Authors:** Qiuwei Li, Peilin Jin, Chenhao Zhao, Renjie Zhang, Cailiang Shen

**Affiliations:** a Department of Orthopedics and Spine Surgery, The First Affiliated Hospital of Anhui Medical University, Hefei, Anhui, China; b Laboratory of Spinal and Spinal Cord Injury Regeneration and Repair, The First Affiliated Hospital of Anhui Medical University, Hefei, Anhui, China

**Keywords:** intervertebral disc degeneration, mechanical stress, notochord cells, single-cell transcriptomics, SPP1-CD44 pathway

## Abstract

**Background:**

Intervertebral disc degeneration (IVDD) is a major cause of spinal disorders, often leading to chronic pain and mobility issues. Mechanical stress is a key factor in IVDD progression, but the underlying mechanisms remain unclear. In this study, we improved a rat intervertebral disc pressure model to explore how mechanical stress affects IVDD, focusing on notochord cell populations and their interactions in the degenerative process.

**Methods:**

We developed a custom pressure device for rats, validated using imaging techniques. Following pressure application, single-cell transcriptomics was employed to analyze dynamic changes in notochord cells in both surgical and sham groups. Gene expression profiles were analyzed for immune regulation, matrix metabolism, and intercellular signaling. We also studied the SPP1 signaling pathway and its interaction with CD44. Finally, we combined Mendelian randomization and human GEO sequencing data to support our results

**Results:**

Pressure application resulted in significant structural damage and abnormal changes in matrix components, worsening over time. Single-cell analysis revealed differences in notochord cell populations between surgical and sham groups, with increased immune regulation and matrix metabolism activity. The SPP1-CD44 signaling pathway was activated in degenerated discs, especially in CD44-expressing cells, underscoring its role in matrix remodeling and inflammation. MR and human GEO sequencing data also support these ideas

**Conclusion:**

This study provides insights into IVDD mechanisms, focusing on the role of the SPP1-CD44 pathway in disc degeneration. We suggest that targeting this pathway may offer potential therapeutic strategies for degenerative spinal diseases.

**HIGHLIGHTS**

- This study developed a modified intervertebral disc degeneration (IVDD) stress model to simulate IVDD by mechanical stress, which provides a more effective platform for studying the mechanism and treatment strategies of IVDD.
- Mechanical stress further aggravates disc degeneration by altering the cell–cell interaction network, especially enhancing immune-related signal transduction.
- During IVDD, notochord cell populations show a high degree of heterogeneity, and the differences in gene expression and function between different subpopulations reveal their different roles in IVDD.
- Activation of the SPP1-CD44 signaling pathway may be a key factor leading to intervertebral disc matrix degradation and activation of the immune response, suggesting that it may be a new therapeutic target.
- The interaction of SPP1 with macrophages enhances the immunomodulatory effect through the CD44 receptor, suggesting that SPP1 plays an important role in regulating the immune environment and inflammatory processes in the intervertebral disc.
- Mendelian randomization and human single-cell sequencing demonstrated the synergistic role of SPP1 and CD44 in IVDD

## Background

Intervertebral disc degeneration (IVDD) is a chronic condition caused by the interaction of multiple factors, and is commonly considered the primary cause of chronic low back pain^[1–3]^. Under physiological conditions, the intervertebral disc is able to dissipate the effects of mechanical loading through the hydrostatic pressure in the nucleus pulposus, while the layered structure of the annulus fibrosus helps maintain mechanical stability^[4,5]^. The initiation of IVDD may be attributed to several factors, including age-related changes in extracellular matrix (ECM) composition, genetic susceptibility, metabolic abnormalities, and abnormal mechanical loading^[6,7]^. Among these, abnormal mechanical pressure is considered a key environmental factor contributing to early disc degeneration, as repetitive heavy lifting in certain occupations has been shown to increase the risk of disc herniation^[8]^. Moreover, studies have confirmed that dynamic compressive loading increases the apoptosis rate of nucleus pulposus cells (NPCs), thereby elevating the risk of disc degeneration^[9]^. We hypothesize that abnormal mechanical pressure influences disc degeneration through the activation of specific cell subpopulations, which play a key role in regulating immune responses and ECM remodeling. We further propose that mechanical stress enhances the activity of these subpopulations, driving the progression of IVDD.

Abnormal mechanical pressure may drive the process of IVDD through dual pathways: short-term loading activates the integrin–focal adhesion kinase (FAK) mechanotransduction pathway, thereby inducing overexpression of matrix metalloprotease (MMP)-3/a disintegrin and metalloproteinase with a thrombospondin motif (ADAMTS)-4^[10]^. In contrast, sustained pressure leads to the invasion of neurovascular structures into the intervertebral disc, resulting in an increase in M1 macrophages, which release inflammatory mediators such as interleukin (IL)-1β and tumor necrosis factor (TNF)-α^[11]^. Notably, notochord cells, as unique embryonic remnant cell populations within the disc, have been found to be mechanically sensitive^[12,13]^. These cells, originating from the notochord structure during embryonic development, remain within the nucleus pulposus during disc development, forming a distinct cell population that constitutes about 30–50% of normal NPCs^[14,15]^. These cells secrete morphogens such as Sonic hedgehog and Noggin, which regulate disc development and matrix homeostasis^[16,17]^. Mechanical stress has long been recognized as a major driver of IVDD, but the specific molecular mechanisms remain insufficiently detailed. The mechanical forces exerted on the intervertebral disc are initially sensed by mechanosensors, such as integrins on the surface of NP and AF cells. These receptors initiate mechanotransduction by converting mechanical stimuli into biochemical signals. The integrin-FAK pathway is a crucial axis in this process, activating downstream signaling events such as PI3K-Akt and MAPK pathways, which regulate cell survival, proliferation, and apoptosis. Prolonged mechanical stress also activates the NF-κB pathway, promoting the production of pro-inflammatory cytokines, including TNF-α, IL-1β, and IL-6, which further contribute to the breakdown of ECM components such as collagen and aggrecan. This inflammatory response accelerates the degenerative process, forming a vicious cycle that worsens disc degeneration. In response to mechanical stress, gene expression within disc cells undergoes significant reprogramming. This interaction amplifies the inflammatory response, further accelerating ECM degradation. Additionally, mechanical loading induces oxidative stress, activating pathways such as ferroptosis and autophagy, which contribute to cell death and matrix turnover, exacerbating the degenerative process. Notochordal cells, which are central to maintaining disc integrity, are particularly sensitive to mechanical stress. Upon loading, notochordal cells undergo phenotypic changes and increase the secretion of morphogens, driving inflammation and matrix degradation. These alterations in notochordal cell function underscore the crucial role of mechanotransduction in disc degeneration.

With the breakthrough of single-cell RNA sequencing (scRNA-seq) technology, unprecedented resolution has been made possible to explore the cellular heterogeneity and molecular regulatory networks of IVDD. Traditional studies, limited by the population-averaging effect of cell or tissue homogenates, are unable to capture the dynamic evolution of specific cell subpopulations, while in contrast, scRNA-seq enables precise identification of functionally-specialized cell subpopulations and the unveiling of their dynamic regulatory networks in the degenerative process^[18–20]^. Previous studies successfully constructed a cellular atlas of the human intervertebral disc using scRNA-seq, identifying eight presumed clusters, comprising chondrocytes, endothelial cells, fibroblasts, macrophages, smooth muscle cells, osteoclasts, proliferating matrix cells, and T cells^[21]^. Further research in rodent models revealed that during degeneration, EGLN3 + StressCs, TGFBR3 + HomCs, and GPRC5A + RegCs exhibit characteristics associated with resistance to mechanical stress, maintenance of internal balance, and repair, respectively. The frequency and features of these cell clusters fluctuate with IVDD. Notably, the chondrogenic differentiation program of protein C receptor-positive progenitor cells is altered by IVDD, while notochord cells transition toward stem cell exhaustion^[22]^. Additionally, CellPhoneDB analysis revealed potential interactions between chondrocytes and other cells in IVDD. One chondrocyte differentiation trajectory interacts with macrophages and endothelial cells, having an amplifying effect on inflammation, which is a critical factor contributing to IVDD^[23]^. During the progression of IVDD, significant intercellular interactions between macrophages and progenitor nucleus pulposus cells occur through the macrophage migration inhibitory factor and nuclear factor (NF)-κB signaling pathways^[24,25]^.

This study, by constructing an improved dynamic pressure model for rat intervertebral discs and integrating single-cell transcriptome sequencing technology, for the first time reveals the core mechanism by which mechanical stress drives IVDD through the “mechanical–immune–metabolic” cascade network mediated by the SPP1 + CD44 + notochord cell subpopulation. Our results revealed that sustained mechanical loading induced expansion of the SPP1 + CD44 + notochord cell subpopulation, which secreted SPP1 protein. This protein, through the CD44 receptor, activated macrophage M1 polarization and triggered a dual-signal cascade: the PI3K–Akt pathway promoted the overexpression of MMP3/ADAMTS5, leading to type II collagen degradation, while the NF-κB pathway induced an IL-6/TNF-α storm, forming an inflammation self-catalysis loop. Single-cell trajectory analysis revealed that the SPP1 + CD44 + subpopulation evolved through the pseudotime axis of “mechanical sensing → immune activation → matrix degradation,” with its gene modules enriched in ECM receptor interactions and ferroptosis-related pathways. Furthermore, experimental results confirmed that high SPP1 expression areas significantly colocalized with macrophage infiltration, lipid peroxidation markers, and downregulated GPX4. The innovative value of this study lies in the novel elucidation of the SPP1–CD44 axis as a mechanical stress transduction hub, which coordinates macrophage polarization and ferroptosis-mediated metabolic remodeling of the intervertebral disc microenvironment. The work has been reported in line with the TITAN criteria^[26]^.

## Materials and methods

### Establishment of the rat IVDD pressure model

Male Sprague–Dawley rats (body weight, 220–250 g) were randomly assigned to two groups of six and anesthetized with 1% pentobarbital sodium (4 mL/kg). The tail vertebrae of the rats were observed using microscopy. The rats were fixed in position, and the positions of Co5 and Co6 were located under X-ray imaging. Puncture points were marked, and the diameter of the tail vertebra was measured using a vernier caliper. The spring compression length was calculated using the formula X = P*m*/k, where *m* represents the cross-sectional area of the cone, k is the spring constant, and P is the pressure (set at a constant 1 MPa). After disinfection and anesthesia, the device was fixed to the tail, and a hammer was used to drive a Kirschner wire through the puncture point, adjusting the spring to the calculated compression length. The spring compression length was calculated based on the formula to ensure precise control of the force applied to the discs. The calibration of pressure was achieved by regularly adjusting the spring to maintain consistent compression length and force during each experiment. To ensure the stability and consistency of the mechanical stimulation, we frequently checked and adjusted the spring compression length before and during the experiments. Each time the device was set up, we measured the spring length and compared it with the precalculated values to ensure that the applied pressure remained within the desired range. This was especially important as even slight variations in the spring length could lead to fluctuations in the applied pressure. After each session, we carefully rechecked the device’s calibration and adjusted the spring tension if necessary to maintain consistent mechanical loading. First 3 days postoperation, the rats received daily intramuscular injections of penicillin. The puncture site was disinfected with iodine tincture and Betadine to prevent infection and ensure sterilization^[27]^. The work has been reported in accordance with the ARRIVE guidelines (Animals in Research: Reporting In Vivo Experiments)^[28]^.

### Identification of the IVDD pressure model

About 1 month after the establishment of the pressure model, the tail vertebrae of the rats were examined using imaging techniques (MRI and X-ray). The rats were then euthanized, and three normal and three model rats were randomly selected for subsequent single-cell sequencing analysis. The remaining rats underwent Western blot (WB), HE staining, and Safranin O-fast green staining to identify IVDD-related markers^[29]^.

### Preparation of single-cell suspensions

Fresh intervertebral disc tissue from the surgically treated rats was carefully dissected to isolate the nucleus pulposus and annulus fibrosus. The nucleus pulposus tissue was preserved in MACS^®^ tissue storage solution until further processing. The samples were washed with phosphate-buffered saline (PBS), cut into small pieces on ice, and then digested with 500 U/mL collagenase I, 150 U/mL collagenase II, 50 U/mL collagenase IV, and 0.1 mg/mL hyaluronidase. After digestion, 3.30 U/mL DNase I and 5% fetal bovine serum (FBS) were added and the mixture was stirred for 60 min at 37°C. The digested tissue was then filtered through a 70 µm cell strainer and centrifuged at 300 × *g* for 5 min. After washing with PBS containing 0.04% BSA, the cell pellets were resuspended in PBS with 0.04% BSA and filtered again through a 35 µm cell strainer^[30]^.

The isolated single cells were stained with Calcein-AM and Draq7 for viability assessment. Dead cells were removed using the MACS Dead Cell Removal Kit to further enrich the single-cell suspension^[31]^.

### Single-cell dimensionality reduction and clustering analysis

The quantitative matrix of the single-cell transcriptome is an MN-dimensional matrix, where each row represents a gene, and each column represents a cell. Typically, a single-cell transcriptome sequencing sample can result in a matrix with tens of thousands of rows (genes) and columns (cells), creating an ultra-high-dimensional dataset. Performing clustering analysis on such high-dimensional data is computationally intensive and challenging for obtaining good clustering results. The large number of dimensions increases data sparsity and introduces noise, making it difficult to detect meaningful patterns. Therefore, dimensionality reduction is generally performed prior to clustering. This process extracts new dimensions from the thousands of genes and represents the data using these new dimensions, which retains as much of the information as possible while reducing data redundancy. By reducing the dimensionality, we minimize the noise and computational burden, which in turn improves the efficiency and accuracy of subsequent clustering operations. Similar cells generally have similar gene expression profiles, so cells with similar expression profiles are clustered together to form a cell group. This step enhances the interpretability of the data and allows for more accurate identification of distinct subpopulations within the tissue. Dimensionality reduction techniques, such as Principal Component Analysis (PCA), t-Distributed Stochastic Neighbor Embedding (t-SNE), or Uniform Manifold Approximation and Projection (UMAP), are commonly used to achieve these goals^[32,33]^.

### Inter-cell cluster similarity analysis

The similarity between cell clusters can be assessed by calculating the Pearson correlation coefficient of the average gene expression between clusters. This analysis is commonly used when identifying cell types or analyzing the functional similarity of cell subtypes. Targeted visualization can be performed for the clusters of interest^[34]^.

### Marker gene identification

Marker genes are defined as genes with high expression in the majority of cells within a specific cell cluster, while being expressed in only a few cells in other clusters. The expression of these genes is significantly upregulated in the given cluster compared to other clusters. The Presto testing method is used to perform differential expression analysis between the targeted cell cluster and the rest of the clusters, with the criteria set as: logfc.threshold greater than 0, and min.pct (the proportion of cells expressing the gene in all cells) greater than 0.25, allowing the identification of marker genes for each cell cluster^[35]^.

### Cell type identification

The SingleR package is used to annotate cells based on public datasets. Due to the limited tissue information and complex origins of the cell types in the dataset, the identified cell types are used as a reference. The final cell-type identification is further validated using known marker genes before proceeding with downstream analysis^[36]^.

### Differential gene expression screening and enrichment analysis

Differentially-expressed genes between groups were identified using the FindMarkers function from the Seurat package. Gene ontology (GO) enrichment analysis, Kyoto Encyclopedia of Genes and Genome (KEGG) pathway enrichment analysis, and protein interaction network analysis were then conducted for the differentially-expressed genes^[37]^.

### Pseudotime analysis

Pseudotime analysis was performed using Monocle2 software. Prior to Monocle analysis, the marker genes from the Seurat clustering results were selected, and the raw expression counts of the cells were filtered. Based on the pseudotime analysis, branch expression analysis modeling analysis was applied to identify genes responsible for determining the branching fate of trajectories^[38]^.

### Cell culture and treatment

To extract notochord cells from the intervertebral disc, the nucleus pulposus was first isolated from rat intervertebral disc tissue. After removal, the tissue was immediately washed with PBS to remove blood and impurities. The tissue was then cut into small pieces, retaining the nucleus pulposus while avoiding damage to the cells. The tissue fragments were placed in a digestion solution containing collagenase (Beyotime Institute of Biotechnology, Jiangsu, China; Cat. No. C5003) and trypsin and incubated at 37°C for 2–4 h. During digestion, the mixture was gently aspirated every 30 min to help release the cells. After digestion, the reaction was stopped by adding medium containing 10% FBS (Gibco/Life Technologies, Carlsbad, CA, USA) and 1% penicillin–streptomycin. The mixture was centrifuged at 400 × *g* for 5 min to remove tissue debris, and the supernatant was collected. The cell suspension was then transferred into medium containing 10% FBS. Next, density gradient centrifugation was performed for further cell separation. After centrifugation, cells were separated into different layers based on their density, with notochord-like cells typically located in the lower-density layers. The notochord-like cell layer was collected, washed to remove residual impurities, and the cells were suspended in appropriate culture medium for further cultivation and verification^[39]^.

### Cell pressure model construction

A custom cell pressurization device was used to apply 1.0 MPa pressure to NPCs for 12, 24, or 48. After pressure treatment, proteins were extracted from the cells and WB was performed to detect degeneration markers (e.g., COL2A, MMP3) and to assess cell proliferation activity using a commercial kit. NPCs were incubated in a no-air environment to exclude the effects of increased CO_2_, which could alter the pH and solubility of the medium due to increased atmospheric pressure^[40]^.

### Fluorescent cell staining

After discarding the culture medium, cells were washed three times with precooled PBS (4°C to minimize cell detachment). The cells were fixed with 4% paraformaldehyde at room temperature for 20 min and permeabilized with 0.3% Triton X-100 for 15 min to enhance antibody penetration. After blocking with 5% donkey serum for 1 h, cells were incubated with primary antibodies: rabbit anti-SPP1 (1:200, Cat. No. AF0227), rabbit anti-CD44 (1:150, Cat. No. AF3570), and rabbit anti-MMP3 (1:100, Cat. No. AF1438) overnight at 4°C in a humidified box to ensure deep cell labeling. The cells were then incubated with the corresponding Alexa Fluor secondary antibodies (594 red/488 green/647 infrared, 1:500) at room temperature for 2 h in the dark. After staining the nuclei with 4′,6-diamidino-2-phenylindole (DAPI) for 5 min and mounting with a hardening agent, images were captured using a fluorescence microscope.

### Immunohistochemical analysis

Caudal vertebrae from rats were decalcified using formaldehyde, dried, embedded in paraffin, and then sectioned. The paraffin-embedded sections were deparaffinized, stained with HE and Safranin O-fast green, dried, mounted on glass slides, and examined under a microscope. Two of the authors evaluated the degree of IVDD in each group.

### Disc height measurement

X-ray images of the rat IVDD were acquired after 8 weeks using a small animal X-ray machine, and the disc height was estimated using ImageJ software (NIH, Bethesda, MD, USA).

### Magnetic resonance imaging

Rats were euthanized 8 weeks after spinal cord puncture under anesthesia with sodium pentobarbital (200 mg/kg). The Co5/6 intervertebral disc structures were examined using a 3.0 T MRI scanner (Bruker BioSpin GmbH, Rheinstetten, Germany), and DICOM radial observation software was used for analysis. The degree of IVDD was assessed using the Pfirrmann grading system^[41]^.

### Tissue immunofluorescence

Tissue samples were prepared for immunofluorescence staining to evaluate the expression of ADAMTS5 (Antibody – DF13268), MMP3 (Antibody – AF0217), aggrecan (Antibody – DF7561), Collagen II (Antibody – AF0135), SPP1 (Antibody – AF0227), CD44 (Antibody – DF6392), ARG1 (Antibody –DF6657), CD206 (Antibody – DF4149), iNOS (Antibody – AF0199), and CD86 (Antibody – DF6332) in the different experimental groups. After deparaffinization and antigen retrieval, the sections were blocked with 5% BSA for 30 min and incubated overnight at 4°C with primary antibodies targeting the proteins of interest. The following day, the sections were washed and incubated with fluorescent-labeled secondary antibodies at room temperature for 1 ho in the dark. Slides were mounted with DAPI-containing mounting medium, and images were captured under a fluorescence microscope. Red fluorescence indicated target protein expression, and blue fluorescence indicated cell nuclei. Fluorescence intensity of each marker was analyzed, and expression levels were compared across groups.

### WB analysis

Cells from each group were separated from their respective culture media. After washing with PBS three times, cells were harvested by scraping to obtain the cell suspension, which was then centrifuged at 1000 rpm for 5 min at 5°C. The cell pellet was collected in an Eppendorf tube. Cells were lysed with a mixture of protease inhibitors, radioimmunoprecipitation assay buffer, and phenylmethylsulfonyl fluoride (Beyotime Institute of Biotechnology). Protein content was measured using a BCA protein assay kit (Beyotime Institute of Biotechnology). The proteins were mixed with loading buffer in a 4:1 ratio and heated for 10 minutes. Proteins were separated by SDS-PAGE and transferred to a Polyvinylidene Fluoride (PVDF) membrane at 120 V. The membrane was then blocked with a quick blocking solution and incubated overnight at 4°C with the following primary antibodies: ADAMTS5 (Antibody – DF13268), MMP3 (Antibody – AF0217), aggrecan (Antibody – DF7561), Collagen II (Antibody – AF0135), SPP1 (Antibody – AF0227), and CD44 (Antibody – DF6392). After incubation with HRP-conjugated secondary antibodies [Rabbit IgG (H + L) HRP-S0001] at room temperature, chemiluminescence was observed using the BeyoECL kit (Beyotime Institute of Biotechnology). Images were captured and analyzed using a multifunctional gel imaging system (Bio-Rad, Hercules, CA, USA).

### Flow cytometry

Notochord cells were cultured under three different conditions: normal group, 500 Pa group (to simulate the stiffness environment of disc degeneration), and 500 Pa + SPP1 inhibition group. To label SPP1 and CD44, PE-labeled anti-SPP1 and anti-CD44 antibodies were used, and cells were incubated at room temperature for 30 min. The cells were then washed with PBS to remove unbound antibodies, resuspended, and prepared for flow cytometric analysis. The cells were detected using a flow cytometer, and PE fluorescence was excited by laser, allowing the system to analyze the expression levels of SPP1 and CD44 on each cell surface. Flow cytometry data were analyzed by setting appropriate thresholds, and the proportion of PE-A positive cells was compared.

### Micro-CT

Rat intervertebral disc samples were removed from the fixative, excess fluid was wiped off with gauze, and the samples were placed on the scanning bed for micro-CT scanning. Images were reconstructed from the raw data using 3D reconstruction software (Recon). Data analysis was performed using Avatar software, with the target region of interest analyzed. All samples were analyzed in the same region to obtain the required parameters and export the data^[42]^.

### MR analysis of SPP1 and CD44 in IVDD

MR analysis was performed to assess the causal relationship between genetic variants in the SPP1 and CD44 genes and IVDD. Genetic variants associated with SPP1 and CD44 were selected from publicly available genome-wide association study (GWAS) databases, specifically using eQTL data for CD44 (eQTL-a-ENSG00000026508) and SPP1 (eQTL-a-ENSG00000118785). The IVDD data were obtained from the DGWAS datasets, namely DGWAS-6924 and DGWAS-1536.

We employed multiple MR methods, including inverse variance weighting (IVW), MR Egger, and the simple median and mode methods, to evaluate the effect of single nucleotide polymorphisms (SNPs) on the expression of SPP1 and CD44 and their association with IVDD. The MR effect size was calculated to quantify the relationship between the selected SNPs and IVDD. To assess the robustness of the causal estimates, statistical significance was determined by examining the confidence intervals and *P* values for the MR estimates. A *P* value less than 0.05 was considered statistically significant. These methods allowed us to draw more accurate conclusions regarding the causal role of these genetic variants in IVDD.

### Statistical analysis

All tests were repeated, with at least three different samples for each condition. Continuous data were reported as mean ± standard deviation. Student’s t-test was used to compare the means of two groups. One-way analysis of variance was performed to compare means among groups, and *P* < 0.05 was considered statistically significant. Statistical graphs were generated using GraphPad Prism 8 software (GraphPad Software Inc., San Diego, CA, USA).

## Results

### Construction of the rat intervertebral disc pressure model

To investigate the mechanisms of IVDD and establish a rat model suitable for this study, we designed and validated an improved intervertebral disc pressure model (Supplemental Digital Content Fig. S1A, available at: http://links.lww.com/JS9/F265). This model simulates the disc degeneration process by applying specific mechanical pressure. Supplemental Digital Content Fig. S1B, available at: http://links.lww.com/JS9/F265 illustrates the pressure model device used in this experiment, along with the results of X-ray imaging (Supplemental Digital Content Fig. S1C, available at: http://links.lww.com/JS9/F265), which revealed significant morphological changes in the intervertebral discs after pressure application, indicating that the model successfully induced disc degeneration. Additionally, X-ray images of rats at 1 week and 1 month postoperation (Supplemental Digital Content Fig. S1D, available at: http://links.lww.com/JS9/F265) showed marked structural changes in the intervertebral discs. The sham operation group exhibited relatively intact disc structures, while the surgical group displayed signs of degeneration, further confirming the model’s validity. These results successfully established the IVDD model and laid the foundation for subsequent studies on the underlying biological mechanisms. Furthermore, we also compared the rats of the two models, namely the acupuncture method and the pressure method. WB found that the pressure model could achieve a better degeneration effect than the acupuncture model (Supplemental Digital Content Fig. S1F-I, available at: http://links.lww.com/JS9/F265).

**Figure 1.**
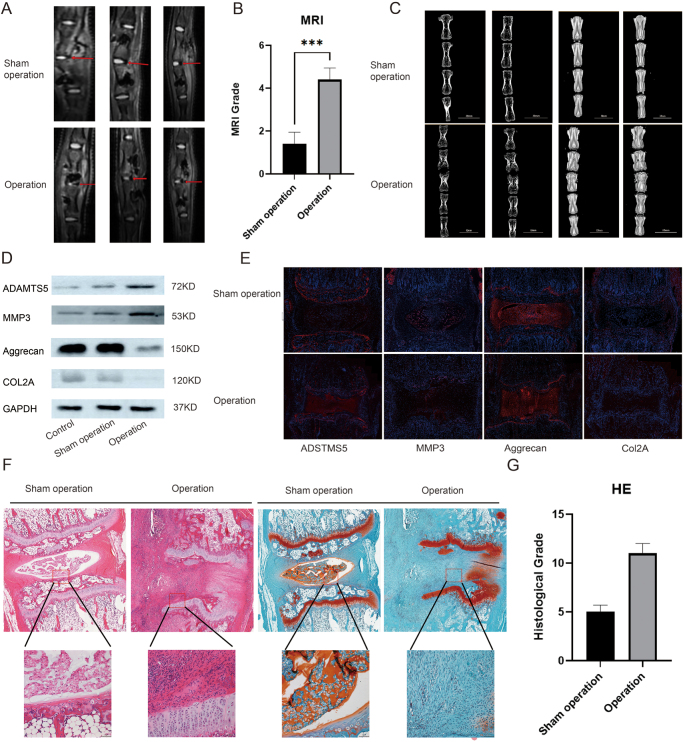
Histological and molecular analysis of pressure-induced disc degeneration. (A) MRI images showing disc changes in the sham operation and surgical groups, with red lines marking areas of degeneration in the surgical group. (B) MRI degeneration grading. (C) X-ray images showing structural changes in the intervertebral discs of both groups, with signs of degeneration in the surgical group. (**D**) WB showing changes in the expression of ADAMTS5, MMP3, aggrecan, and COL2A in the surgical group. **E.** Immunofluorescence staining showing expression of ADAMTS5, MMP3, aggrecan, and COL2A, with red fluorescence indicating protein expression and blue representing nuclei. (F) Tissue section staining showing morphological changes in the discs, with HE staining for structural changes and Alcian blue staining for cartilage metabolic changes. (G) HE grade score.

To further verify the molecular mechanisms of IVDD, we analyzed disc changes in the surgical and sham operation groups using MRI, X-ray imaging, WB, immunofluorescence staining, and tissue section staining. MRI and X-ray imaging results showed obvious signs of degeneration in the intervertebral discs of the surgical group, such as reduced disc height and signal changes (Fig. 1A and B). WB analysis further revealed changes in the expression of molecular markers, such as ADAMTS5, MMP3, aggrecan, and COL2A in the surgical group, indicating significant alterations in regulation of these key proteins during disc degeneration (Fig. 1C). Immunofluorescence staining also showed a significant increase in the expression of ADAMTS5 and MMP3, while the expression of aggrecan and COL2A was reduced in the surgical group (Fig. 1D). Moreover, tissue section staining (HE and Alcian blue staining) revealed structural damage and alterations in the cartilage matrix of the intervertebral discs in the surgical group, further validating the pressure-induced disc degeneration model (Fig. 1E). These results suggest that mechanical pressure significantly accelerates the disc degeneration process and participates in it through modulation of related molecular markers.

### Single-cell transcriptomic analysis of the pressure-induced IVDD model

**Figure 2.**
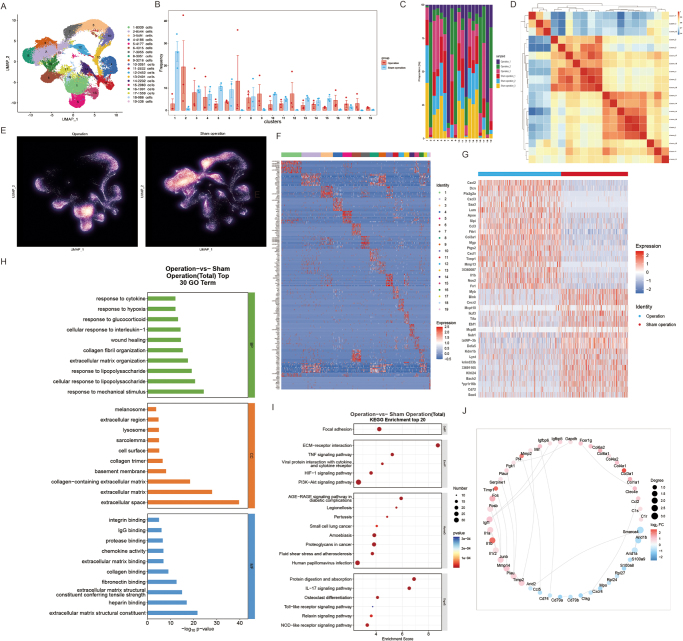
Single-cell transcriptomic analysis of disc degeneration in surgical and sham operation groups. (A) UMAP plot showing cell clustering in the surgical and sham operation groups. (B) Proportional changes in cell populations between groups. (C) Heatmap of cell population distribution in both groups. (D) Heatmap showing correlations between cell populations. **(E)** UMAP plot highlighting gene expression differences between groups. (F) Heatmap of gene expression patterns in cell populations. (G) Differential gene expression between the groups. **H**. GO enrichment analysis of differentially-expressed genes. **I**. KEGG pathway enrichment analysis of top pathways. **J**. Intercellular interaction network analysis between groups.

We performed scRNA-seq analysis on the intervertebral discs of rats in the surgical and sham operation groups to explore transcriptomic changes at the cellular level during disc degeneration. The violin graphs of the number of genes, the number of UMI, the proportion of the number of genes per unit UMI (log10GenesPerUMI), the proportion of mitochondrial UMI (percent_mito), and the proportion of red blood cell genes (percent_HB) of each cell before and after quality control are supplemented as follows, as shown in Supplemental Digital Content Fig. S2, available at: http://links.lww.com/JS9/F265. UMAP analysis revealed significant differences in cell population distribution between the surgical and sham groups (Fig. 2A). The proportional changes in cell populations further confirmed this, with certain cell populations showing a significant increase in the surgical group (Fig. 2B). Heatmap analysis revealed the distribution characteristics and correlations between these cell populations (Fig. 2C and D). Comparison of gene expression patterns showed a significant upregulation of certain key genes in the surgical group (Fig. 2E and F). GO enrichment analysis indicated that differentially-expressed genes in the surgical group were primarily enriched in biological processes related to cytokine response, response to external stimuli, and mechanical stress response (Fig. 2H). KEGG pathway enrichment analysis further revealed significant enrichment of pathways associated with disc degeneration, such as ECM–receptor interaction, PI3K–Akt, and NF-κB signaling pathways, in the surgical group (Fig. 2I). Additionally, intercellular interaction network analysis indicated that key genes in the surgical group play central roles in cell-to-cell interactions (Fig. 2J). These results identify significant cellular and molecular changes in the disc degeneration process induced by surgery, revealing the potential mechanisms underlying relevant signaling pathways and gene interactions.

In this study, we identified and named several distinct cell populations within the intervertebral disc, based on single-cell transcriptomic analysis. The cells were categorized as follows: T/NK cells were identified in cluster 14; B cells in clusters 1, 5, 15, and 18; myeloid cells in clusters 4 and 12; neutrophils in clusters 3, 7, 10, and 13; endothelial cells in cluster 19; dendritic cells (DCs) in cluster 11; notochord cells in clusters 6 and 16; nucleus pulposus progenitor cells (NPPC) in cluster 9; and chondrocytes in clusters 2, 8, and 17. This classification highlights the diverse immune, progenitor, and structural cell populations involved in the intervertebral disc and their potential roles in disc homeostasis and degeneration.

### Single-cell transcriptomic analysis of the notochord subpopulation

We further performed single-cell transcriptomic analysis of the notochord subpopulation in the surgical and sham operation groups. UMAP analysis revealed significant differences in the distribution of notochord cell populations between the two groups, indicating that the surgical treatment affected the composition of the notochord cell population (Supplemental Digital Content Fig. S3A, available at: http://links.lww.com/JS9/F265). Bar graphs showed proportional changes in the different cell populations, with certain populations in the surgical group significantly increased, reflecting an increase in immune-related cells during the degeneration process (Supplemental Digital Content Fig. S3B, available at: http://links.lww.com/JS9/F265). Heatmap analysis further revealed changes in the correlation and expression patterns between these populations (Supplemental Digital Content Fig. S3C, available at: http://links.lww.com/JS9/F265, Supplemental Digital Content Fig. S3D, available at: http://links.lww.com/JS9/F265), with significant changes in the expression of multiple genes in the surgical group, particularly in the notochord cell populations, suggesting that these changes may be related to the disc degeneration process (Supplemental Digital Content Fig. S3E, available at: http://links.lww.com/JS9/F265). Furthermore, KEGG pathway enrichment analysis (Supplemental Digital Content Fig. S3G, available at: http://links.lww.com/JS9/F265) and GO analysis (Supplemental Digital Content Fig. S3H, available at: http://links.lww.com/JS9/F265) revealed key signaling pathways and biological processes within these cell populations, including immune response, matrix degradation, and cell migration. Overall, these results indicate that the disc degeneration process is accompanied by significant changes in the distribution and function of notochord subpopulations, particularly in the activation of inflammation and immune-related pathways. We identified 12 distinct subpopulations based on specific marker genes and functional roles: inflammatory modulating notochordal cells (IMNC), cholesterol metabolism notochordal cells (CMNC), neuroregulatory notochordal cells (NRNC), immune responsive notochordal cells (IRNC), neutrophil associated notochordal cells (NANC), stress responsive notochordal cells (SRNC), matrix forming notochordal cells (MFNC), apoptosis regulating notochordal cells, smooth muscle-like notochordal cells (SMNC), B cell-like notochordal cells, endothelial-like notochordal cells, and cartilage matrix notochordal cells (CMNC). These subpopulations were defined based on differential expression of specific marker genes and functional annotations related to cell signaling, matrix remodeling, immune responses, and metabolic processes. For example, IMNC were identified by the expression of inflammatory mediators, while MFNC were characterized by their role in ECM production. The naming reflects the primary functional roles of these cells in maintaining disc homeostasis or contributing to degeneration. These findings reveal the diverse roles of notochord subpopulations in disc degeneration and provide insights for potential therapeutic targets. Furthermore, in order to prove the reliability of our naming of the 12 subpopulations of chordal cells, we performed immunofluorescence staining using the key top marker genes of CMNC and NRNC. The staining results demonstrated that there was no significant difference in the expression of the CMNC subpopulation between operation and sham operation. The NRNC subgroup was significantly expressed in operation compared with the sham operation group (Supplemental Digital Content Fig. S3I, available at: http://links.lww.com/JS9/F265).

### Transcriptomic profiling and functional analysis of notochordal subpopulations in disc degeneration

**Figure 3.**
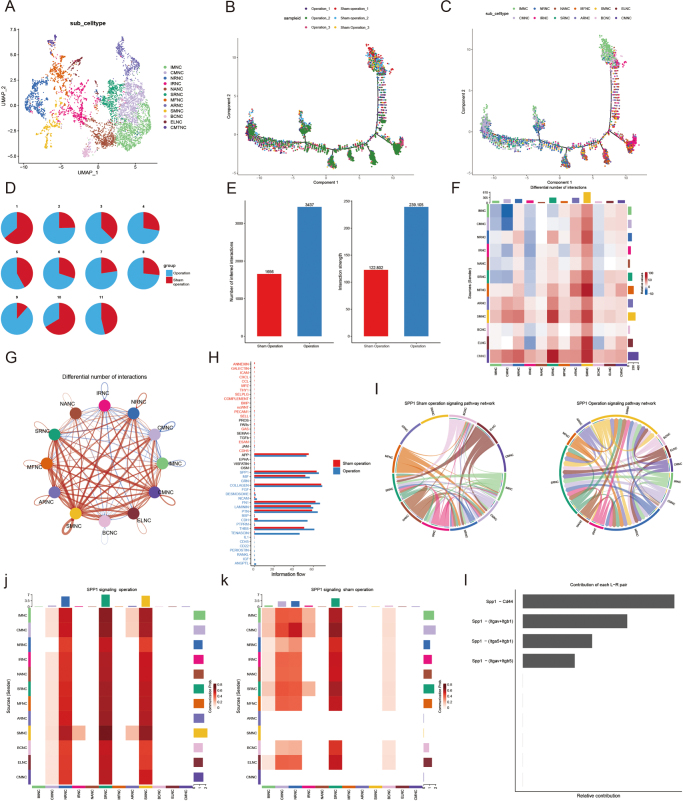
Transcriptomic features and cell interaction analysis of notochord subpopulations. A. UMAP plot showing notochord cell subpopulations in the surgical and sham operation groups. B. Distribution differences of cell populations between the two groups. C. UMAP trajectory analysis of cell evolution in different states. D. Proportional changes in cell populations between the surgical and sham groups. E. Analysis of differentially-expressed genes and transcript length differences. F. Heatmap showing the interaction strength between cell populations in both groups. G. Network of differential intercellular interactions. H. Differences in immune-related information flow between the groups. I. Expression differences in the SPP1 signaling pathway. J. Expression pattern differences of notochord cells in the SPP1 pathway. K. Differences in SPP1 pathway intensity between groups. L. Contribution of each cell population to the SPP1 signaling pathway.

We analyzed the transcriptomic features and intercellular interactions of notochordal subpopulations in the intervertebral discs of rats from the surgical and sham operation groups. UMAP analysis (Fig. 3A) revealed clear differences in notochord cell populations between the two groups, with distinct cell distributions and evolutionary trajectories (Fig. 3B and C). These differences were further confirmed by varying cell population proportions, with certain populations being more abundant in the surgical group (Fig. 3D). Gene expression analysis (Fig. 3E) indicated significant changes between the surgical and sham operation groups, particularly in immune-related pathways. Intercellular communication analysis (Fig. 3F) highlighted immune signaling differences, underscoring the role of immune pathways in disc degeneration. The SPP1 signaling pathway was significantly upregulated in the surgical group (Fig. 3I), particularly within immune-related subpopulations such as IRNC, IMNC, and NANC (Fig. 3J and K). Cell population contributions to this pathway (Fig. 3L) revealed that MFNC, IMNC, and NANC were central regulators of the SPP1 signaling axis.

To investigate further, we analyzed the expression patterns of SPP1 and CD44 across notochordal subpopulations using pseudotime trajectory analysis. Temporal changes in cell populations and sequential gene expression alterations were observed (Supplemental Digital Content Fig. S4A, B, available at: http://links.lww.com/JS9/F265). The gene expression heatmap (Supplemental Digital Content Fig. S4C, available at: http://links.lww.com/JS9/F265) demonstrated notable differences in SPP1 and CD44 expression, particularly in IRNC and IMNC (Supplemental Digital Content Fig. S4D, available at: http://links.lww.com/JS9/F265). KEGG and GO enrichment analysis of different gene modules identified pathways associated with disc degeneration, including autophagy, endotoxin response, and cell adhesion molecule pathways (Supplemental Digital Content Fig. S5, available at: http://links.lww.com/JS9/F265). Specifically, module 2 was enriched in pathways related to oxidative phosphorylation, cell signaling, and cancer, suggesting their involvement in disc degeneration. Correlation analysis (Supplemental Digital Content Fig. S4E, available at: http://links.lww.com/JS9/F265) revealed a positive association between SPP1 and CD44 expression in IRNC and IMNC, supporting their cooperative role in disc degeneration. Violin plots further showed significant differences in SPP1 and CD44 expression between the surgical and sham operation groups, with increased expression in subpopulations like MFNC and IRNC in the surgical group (Supplemental Digital Content Fig. S4F and G, available at: http://links.lww.com/JS9/F265), indicating their importance in the degeneration process.

### Transcriptomic and functional analysis of notochordal and myeloid cell interactions in disc degeneration

**Figure 4.**
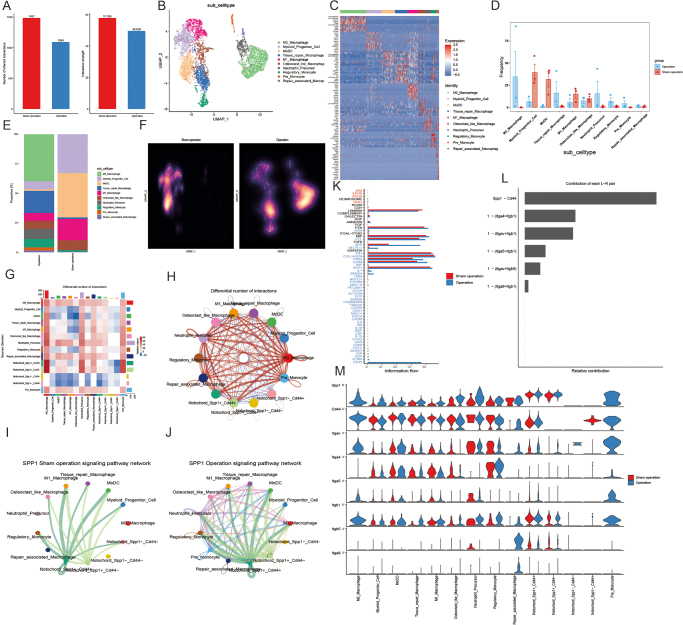
Interaction analysis of SPP1 + CD44 + Notochord cells with immune cells. (A) Proportional changes in myeloid subpopulations between surgical and sham groups. (B) UMAP plot of the distribution of myeloid cell populations in both groups. (C). Heatmap showing gene expression differences between myeloid cell populations. (D) Frequency changes of myeloid cell populations in surgical and sham groups. (E) Proportional changes in myeloid cells between groups. F. UMAP plot showing coexpression of notochord cells with myeloid cells. (G) Heatmap of intercellular information flow, showing strong interaction between notochord cells and myeloid cells. (H) Differential interaction network highlighting key signaling pathways between notochord cells and myeloid cells. (I) Immune regulatory network of SPP1 signaling, showing cooperation between notochord and myeloid cells in immune responses. (J) Differences in SPP1 signaling pathway expression in myeloid cells between groups. (K) Differences in immune information flow, showing interaction intensity between myeloid and notochord cells in both groups. (L) Contribution analysis of notochord cells and myeloid cells in signal transduction. (M) Violin plots showing expression changes of SPP1 and CD44 in notochord cells between surgical and sham groups.

We analyzed the transcriptomic features of notochordal subpopulations in the intervertebral discs of rats from the surgical and sham operation groups, focusing on the expression of SPP1 and CD44 and their role in immune response and ECM remodeling. Based on different expression patterns of Spp1 and Cd44, we divided notochordal cells into four subpopulations: Spp1 + Cd44 +, Spp1 + Cd44 −, Spp1 − Cd44 +, and Spp1 − Cd44 −, which allowed us to investigate their distinct functional roles. UMAP analysis (Supplemental Digital Content Fig. S6A, available at: http://links.lww.com/JS9/F265) revealed significant differences in cell coexpression between the surgical and sham groups, particularly in specific notochordal subpopulations (Supplemental Digital Content Fig. S6B, available at: http://links.lww.com/JS9/F265). Gene expression analysis (Supplemental Digital Content Fig. S6C and D, available at: http://links.lww.com/JS9/F265) showed notable differences in genes related to immune response (e.g., Wnt signaling, Ccl18, Cxcl5) and ECM remodeling (Supplemental Digital Content Fig. S6F, available at: http://links.lww.com/JS9/F265), with an increased proportion of relevant cell populations in the surgical group (Supplemental Digital Content Fig. S6E, available at: http://links.lww.com/JS9/F265). Violin plots confirmed these expression differences, particularly in immune and ECM-related genes (Supplemental Digital Content Fig. S6H, available at: http://links.lww.com/JS9/F265), and GO and KEGG enrichment analyses revealed key roles of these genes in regulating immune responses and ECM remodeling during disc degeneration (Supplemental Digital Content Fig. S6I and J, available at: http://links.lww.com/JS9/F265). These results suggest that the coexpression of SPP1 and CD44 plays a crucial role in modulating immune responses and ECM remodeling in the context of disc degeneration. To verify the key Spp1 + Cd44 + cluster, we employed immunofluorescence techniques and conducted verification using triple staining with SPP1, CD44, and Brachyury antibodies. The results are shown in Figure 4K. More Spp1 + Cd44 + cell clusters appeared in the surgical group compared with the sham operation group (Supplemental Digital Content Fig. S6K, available at: http://links.lww.com/JS9/F265).

Further investigation into myeloid cell interactions revealed 10 distinct subpopulations, including M1 and M2 macrophages, tissue-repair macrophages, and monocyte-derived dendritic cells (MoDCs) (Fig. 4B and C). Quantitative comparisons showed a significant increase in myeloid cell abundance in the surgical group (Fig. 4D), with a shift toward a pro-inflammatory phenotype. The fraction of M1 macrophages increased significantly, while M2 macrophages (antiinflammatory and tissue-reparative) were reduced. Ligand-receptor interaction analysis (Fig. 4G and H) revealed extensive cross-talk between SPP1 + CD44 + notochordal cells and myeloid cells, particularly pro-inflammatory myeloid subtypes such as M1 macrophages and MoDCs. The Spp1 + Cd44 + notochordal subpopulation emerged as a key hub for these interactions, showing the highest predicted interaction strength with immune cells. Gene expression analysis confirmed that this subpopulation uniquely coexpresses high levels of Spp1 and Cd44 (Fig. 4M), and network diagrams of cell–cell communication indicated that SPP1 signaling was greatly enhanced in the surgical group (Fig. 4I and J). Contribution analysis further confirmed that SPP1 signaling plays a dominant role in notochordal–myeloid communication following disc injury. Together, these findings highlight the dynamic and coordinated cell–cell interactions between notochordal and myeloid cells in disc degeneration, particularly through SPP1–CD44 signaling, which may provide new therapeutic targets for modulating the immune response and ECM remodeling in disc degeneration.

### SPP1 + CD44 + Notochord cell interactions with macrophages in disc degeneration

We analyzed the interactions between SPP1 + CD44 + notochordal cells and macrophages in the context of disc degeneration. Pseudotime trajectory analysis (Supplemental Digital Content Fig. S7A, available at: http://links.lww.com/JS9/F265 and Supplemental Digital Content Fig. S7B, available at: http://links.lww.com/JS9/F265) revealed significant dynamic changes in gene expression along the degenerative process, particularly in macrophage-related genes such as those involved in inflammation and immune response (Supplemental Digital Content Fig. S7C, available at: http://links.lww.com/JS9/F265). GO and KEGG pathway enrichment analyses (Supplemental Digital Content Fig. S8, available at: http://links.lww.com/JS9/F265) highlighted several crucial pathways involved in disc degeneration, including cell cycle regulation, ECM remodeling, and metabolic processes, all of which are integral to the inflammatory response and tissue degradation observed in the operation group.

Evolutionary trajectories (Supplemental Digital Content Fig. S7D, available at: http://links.lww.com/JS9/F265 and Supplemental Digital Content Fig. S7E, available at: http://links.lww.com/JS9/F265) demonstrated that SPP1 + CD44 + notochordal cells are closely linked to M1_Macrophages (pro-inflammatory) and M2_Macrophages (tissue repair), highlighting their pivotal role in immune regulation during degeneration. The SPP1 + CD44 + notochordal cells, with their elevated expression of SPP1 and CD44, were particularly active in interacting with macrophages, as shown by their strong association with both M1 and M2 macrophage subpopulations. These interactions suggest a coordinated response between notochordal cells and macrophages, where SPP1 + CD44 + notochordal cells may promote inflammation and tissue degradation through M1 macrophage activation, while also influencing repair processes via M2 macrophages.

Violin plot analyses (Supplemental Digital Content Fig. S7F, available at: http://links.lww.com/JS9/F265 and Supplemental Digital Content Fig. S7G, available at: http://links.lww.com/JS9/F265) confirmed significant differences in the expression of SPP1 and CD44 between the surgical and sham groups, with SPP1 + CD44 + notochordal cells showing a marked increase in the surgical group. This suggests that the injury-induced inflammation promotes the activation of these cells. Moreover, correlation analysis (Fig. 7H and 7I) revealed a positive correlation between SPP1 + CD44 + notochordal cells and both M1_Macrophages and M2_Macrophages, further supporting the notion that these notochordal cells play a central role in modulating immune responses during disc degeneration, contributing to both inflammation and tissue repair.

### SPP1 + CD44 + Notochord cell interactions with macrophages and matrix gene expression in disc degeneration

To investigate the relationship between SPP1 + CD44 + notochordal cells and macrophages in disc degeneration, we analyzed gene expression and intercellular interactions. Pseudotime analysis revealed dynamic changes in the expression of key genes such as SPP1, COL1A1, COL2A1, and Laminin across macrophage states (Supplemental Digital Content Fig. S9A–C, available at: http://links.lww.com/JS9/F265). SPP1 was significantly upregulated in both notochordal cells and macrophages in the surgical group, suggesting its central role in matrix degradation and immune regulation during degeneration. The expression of these genes varied across macrophage states, with M1 macrophages showing higher levels of SPP1 and Laminin, while M2 macrophages demonstrated elevated COL1A1 and COL2A1 expression, indicative of a shift toward tissue repair (Supplemental Digital Content Fig. S9D–J, available at: http://links.lww.com/JS9/F265).

Further analysis (Supplemental Digital Content Fig. S9, available at: http://links.lww.com/JS9/F265) revealed a strong positive correlation between SPP1 + CD44 + notochordal cells and macrophages, particularly in the context of immune response and matrix remodeling. SPP1 + CD44 + notochordal cells showed significant coexpression with pro-inflammatory M1 macrophages (Supplemental Digital Content Fig. S9K and N, available at: http://links.lww.com/JS9/F265), supporting the hypothesis that SPP1 + CD44 + notochordal cells may drive the activation of macrophages through the SPP1-CD44 signaling axis. In turn, M1 macrophages promoted inflammation and matrix degradation, while M2 macrophages were more involved in repair processes, contributing to ECM remodeling in response to SPP1 + CD44 + notochordal cell signaling.

Violin plot analysis further confirmed the enhanced expression of immune-related genes (e.g., Ccl2, Cxcl10, IL-10, and Tgfβ1) in SPP1 + CD44 + notochordal cells compared to other notochordal subpopulations (Supplemental Digital Content Fig. S10A–F, available at: http://links.lww.com/JS9/F265), reinforcing their role in regulating macrophage polarization and the inflammatory response. These findings suggest that SPP1 + CD44 + notochordal cells are crucial in orchestrating immune responses, influencing both M1 and M2 macrophages, and driving the progression of disc degeneration.

### Effects of different pressures on notochord cells and expression of SPP1 and CD44

**Figure 5.**
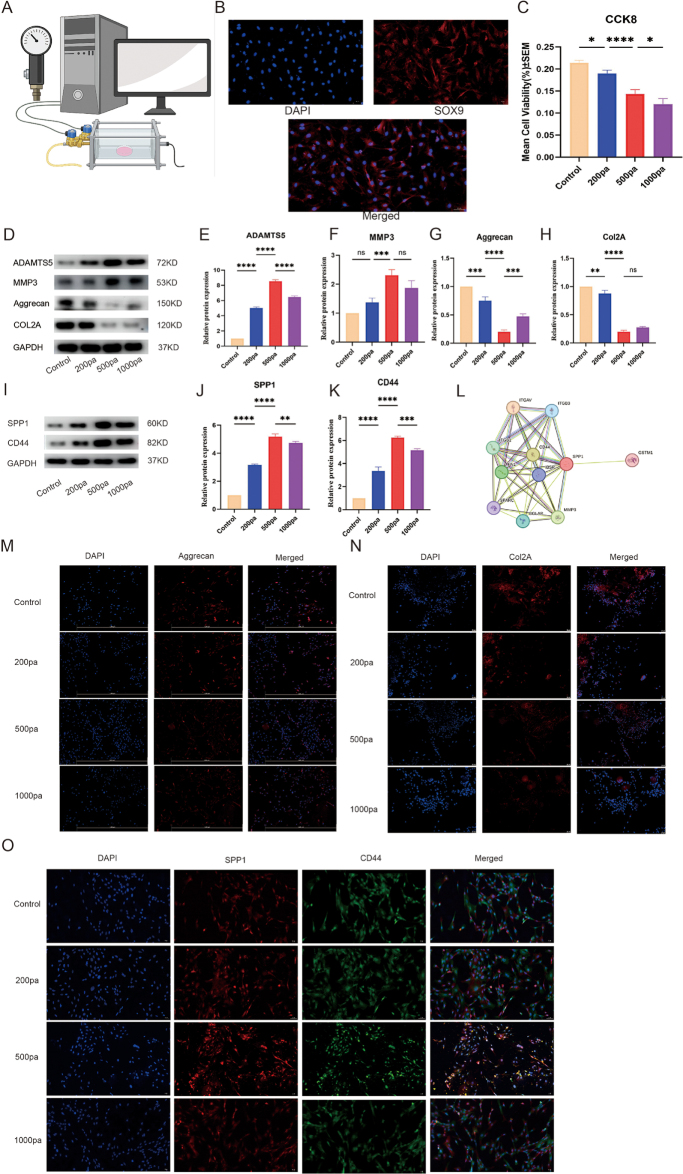
Effects of different pressures on notochord cells and expression of SPP1 and CD44. (A) Experimental design showing different pressure conditions applied to notochord cells. (B) Fluorescence identification of notochord cells. (C) CCK-8 assay results showing the effect of different pressure conditions on cell activity. (D) WB analysis showing expression changes of ADAMTS5, MMP3, aggrecan, and COL2A under different pressure conditions. (E) Relative protein expression changes of ADAMTS5 under different pressures. (F) Relative protein expression changes of MMP3 under different pressures. G. Relative protein expression changes of aggrecan under different pressures. (H) Relative protein expression changes of COL2A under different pressures. (I) WB analysis showing changes in SPP1 and CD44 protein expression. (J) Relative protein expression changes of SPP1 under different pressures. (K) Relative protein expression changes of CD44 under different pressures. L. PPI network highlighting SPP1 interactions with matrix and immune-related proteins. (M) Immunofluorescence staining focusing on aggrecan expression. (N) Immunofluorescence staining focusing on COL2A expression. (O) Immunofluorescence double staining showing changes in SPP1 and CD44 expression under different pressures.

To investigate the effects of different pressure conditions on the morphology and gene expression of notochordal cells, we employed a cell pressure system (Fig. 5A). Notochordal cells were identified using Sox9 and COL2A double staining, confirming their presence and identity (Fig. 5B). CCK-8 assay results (Fig. 5C) indicated a significant decrease in cell activity with increasing pressure, with the lowest activity observed in the 1000 Pa group, suggesting that elevated pressure has a detrimental effect on notochordal cell viability.

WB analysis (Fig. 5D) revealed significant changes in the expression of key genes involved in matrix remodeling, including ADAMTS5, MMP3, aggrecan, and COL2A, under different pressure conditions. Specifically, the 500 Pa and 1000 Pa groups showed significant alterations in the expression of these genes (Fig. 5E–H), indicating that higher pressure levels strongly influence the degradation and synthesis of ECM components in notochordal cells, which aligns with the pressure-induced disc degeneration observed.

Additionally, WB analysis of SPP1 and CD44 expression (Fig. 5I–K) revealed a significant increase in both proteins as pressure increased, particularly in the 500 Pa and 1000 Pa groups. These results suggest that SPP1 and CD44, key molecules identified in previous analyses (such as the interaction between SPP1 + CD44 + notochordal cells and macrophages), may play a pivotal role in mediating pressure-induced disc degeneration by modulating both immune responses and matrix degradation. PPI analysis identified SPP1 as a central hub interacting with proteins involved in matrix remodeling, immune modulation, and signaling, suggesting its key role in disc degeneration (Fig. 5L).

Immunofluorescence staining (Fig. 5M and O) further confirmed the upregulation of matrix proteins, such as aggrecan and COL2A, along with SPP1 and CD44, under higher pressure conditions. This supports the hypothesis that these molecules are involved in pressure-induced changes in the disc microenvironment, likely contributing to the degeneration process through both matrix degradation and immune modulation, as highlighted in earlier findings with SPP1 + CD44 + notochordal cells.

### Effects of different pressures on notochord cells and the role of SPP1 inhibition

**Figure 6.**
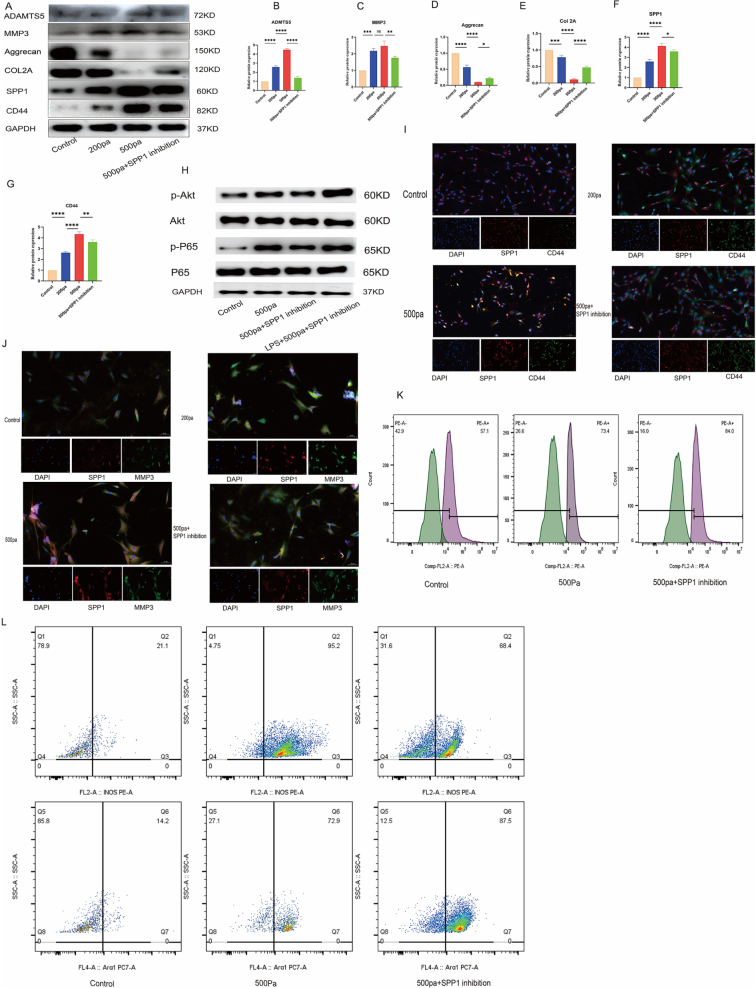
Effects of different pressures on notochord cells and the role of SPP1 Inhibition. (A) WB analysis of different proteins under varying pressure conditions. (B–E) Relative protein expression levels of SPP1, CD44, aggrecan, and COL2A in different experimental groups. (F, G) WB analysis of SPP1 and CD44 in notochord cells under different pressures. (H) WB analysis was performed to evaluate the activation of the Akt and NF-κB pathways in different treatment groups. (I) Immunofluorescence analysis showing changes in SPP1 and CD44 expression under different pressure conditions. (J) Colocalization analysis of SPP1 and MMP3 under 500 Pa pressure. (K) Flow cytometry results showing CD44 expression under different pressure conditions. (L) Analysis of macrophage polarization by flow cytometry.

To investigate the effects of mechanical pressure on disc degeneration, we utilized a 500 Pa pressure model to simulate degenerative conditions and assessed cellular responses using WB, IF, and flow cytometry. Initially, we evaluated the protein expression levels of ADAMTS5, MMP3, aggrecan, COL2A, SPP1, and CD44 under varying pressure conditions (Fig. 6A). The results demonstrated that exposure to 500 Pa significantly increased the expression of SPP1 and CD44, while inhibition of SPP1 reduced their expression, supporting the role of SPP1 + CD44 + notochordal cells in mediating cellular responses during disc degeneration.

**Figure 7.**
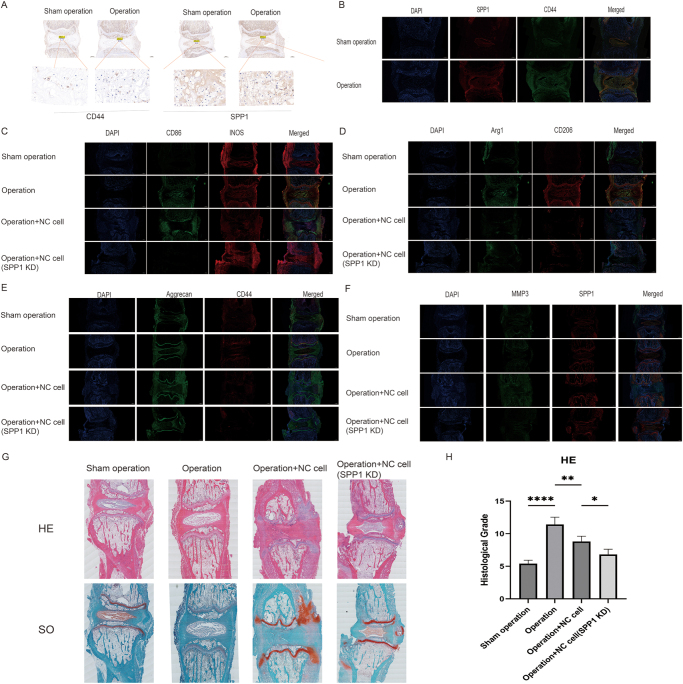
Effect of SPP1 on immune response and macrophage polarization in notochord cells. A. Immunohistochemical staining showing increased expression of CD44 and SPP1 in the surgical group. B. Immunofluorescence staining showing coexpression of SPP1 and CD44, with higher expression in the surgical group. C. Immunofluorescence staining showing increased CD86 and iNOS expression in the surgical group, indicating inflammation. D. Immunofluorescence staining showing Arg1 and CD206 expression changes in different groups, indicating macrophage polarization. E. Immunofluorescence staining, aggrecan and CD44 expression. F. Immunofluorescence staining showed that SPP1 and MMP3 were colocalized. G. HE and Safranin O staining showed disc tissue degeneration. H. HE histological score.

Quantitative analysis (Fig. 6B–E) revealed that under 500 Pa pressure, SPP1 and CD44 were significantly upregulated, while aggrecan and COL2A expression was downregulated, indicating pressure-induced matrix degradation. However, SPP1 inhibition partially restored the expression of aggrecan and COL2A, highlighting that SPP1 plays a critical role in regulating matrix synthesis and degradation in response to mechanical stress. This aligns with previous findings suggesting that SPP1 + CD44 + notochordal cells are central to immune modulation and ECM remodeling in disc degeneration.

WB analysis further confirmed that SPP1 inhibition effectively reduced the expression of both SPP1 and CD44 (Fig. 6F and G). WB analysis was performed to evaluate the activation of the Akt and NF-κB pathways in different treatment groups. The results showed that 500 Pa pressure treatment increased the phosphorylation of both Akt and P65 compared to the control group, indicating activation of these pathways. In the 500 Pa + SPP1 inhibition group, phosphorylation of Akt and P65 was reduced, suggesting that SPP1 inhibition attenuated the activation of these pathways. Additionally, the LPS + 500 Pa + SPP1 inhibition group showed a similar reduction in phosphorylation, reinforcing the role of SPP1 in modulating immune signaling. GAPDH served as the loading control. Immunofluorescence staining (Fig. 6I) showed that SPP1 and CD44 expression was higher under 500 Pa pressure, which decreased with SPP1 inhibition, further supporting the involvement of SPP1 in the cellular response to pressure-induced degeneration. Colocalization analysis (Fig. 6J) revealed that SPP1 coexpressed with MMP3 under 500 Pa, and SPP1 inhibition reduced MMP3 expression, suggesting that SPP1 regulates matrix degradation through MMP3, which is a key player in ECM turnover.

Finally, flow cytometry (Fig. 6K) demonstrated a significant increase in CD44 expression under 500 Pa pressure. Inhibition of SPP1 effectively reduced CD44 expression, indicating that SPP1 is involved in ECM remodeling and intercellular signaling via CD44. These findings confirm that SPP1 + CD44 + notochordal cells play a pivotal role in mediating ECM degradation and immune responses during disc degeneration, potentially contributing to the progression of disc degeneration under mechanical stress. We explored the interaction between chordal cells and macrophages under different treatments and their impact on macrophage polarization. The control group, consisting of normal chordal cells, showed baseline macrophage polarization. In the second group, 500 Pa pressure-treated notochord cells cocultured with macrophages promoted M1 polarization, indicating that mechanical pressure enhanced the pro-inflammatory response through notochord cells. The third group, with 500 Pa + SPP1 inhibition-treated notochord cells, showed reduced M1 and increased M2 macrophages, suggesting that SPP1 inhibition partially reversed the stress-induced immune response and promoted M2 polarization (Fig. 6L).

### Effect of SPP1 on immune response and macrophage polarization in notochord cells

To validate the role of SPP1 from single-cell transcriptomic analysis, we examined the expression of SPP1 and CD44 in the surgical and sham operation groups using immunohistochemistry and immunofluorescence staining. The results revealed a significant increase in the expression of SPP1 and CD44 in the surgical group (Fig. 7A and B), consistent with our transcriptomic analysis indicating immune response activation. This upregulation of SPP1 and CD44 highlights their involvement in the inflammatory microenvironment during disc degeneration.

Further analysis of macrophage polarization revealed that the surgical group exhibited increased expression of M1 markers (CD86 and iNOS) and decreased expression of M2 markers (Arg1 and CD206) (Fig. 7C and D), indicating a shift toward an inflammatory response. Notably, SPP1 inhibition resulted in a shift in macrophage polarization toward the M2 phenotype, suggesting that SPP1 plays a crucial role in regulating macrophage polarization during disc degeneration (Fig. 7D).

To investigate the effects of SPP1 inhibition on ECM remodeling, we analyzed the expression of aggrecan and CD44 in the pressure model. Immunofluorescence staining (Fig. 7E) showed a significant reduction in aggrecan expression in disc cells under the pressure model, which was partially restored upon SPP1 inhibition. This suggests that SPP1 contributes to ECM degradation in response to mechanical stress. Furthermore, colocalization analysis of SPP1 and MMP3 revealed that SPP1 coexpression with MMP3 was significantly reduced in the SPP1 inhibition group under the pressure model (Fig. 7F). This indicates that SPP1 regulates matrix degradation by modulating MMP3 expression, and its inhibition reduces matrix degradation, offering insight into potential therapeutic mechanisms for disc degeneration.

Histological analysis using HE and Safranin O staining (Fig. 7G) revealed that the pathological changes in intervertebral disc tissue were more severe under the pressure model. However, SPP1 inhibition alleviated histological damage to some extent, suggesting that blocking SPP1 may mitigate the effects of mechanical stress on disc tissue. Histological scoring of HE staining (Fig. 7H) further confirmed that the SPP1 inhibition group exhibited significantly lower scores compared to both the operation and sham operation groups, indicating that SPP1 inhibition effectively alleviates IVDD under the pressure model.

### SPP1 regulation of disc degeneration and imaging and biomarker analysis

**Figure 8.**
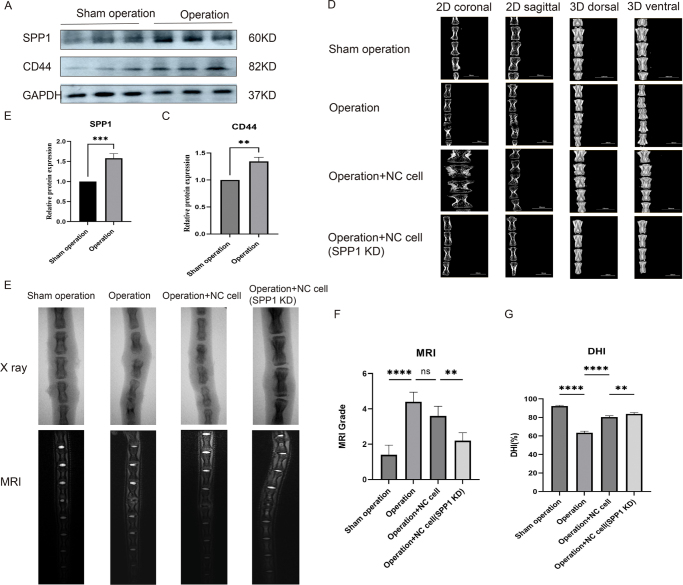
Role of SPP1 in disc degeneration and imaging evaluation. (A) WB analysis of SPP1 and CD44 protein expression in the surgical and sham operation groups. (B) Relative protein expression of SPP1. (C) Relative protein expression of CD44. (D) Imaging evaluation showing changes in disc structure in the different groups using 2D coronal, 2D sagittal, and 3D dorsal and ventral images. (E) X-ray imaging and MRI showing disc changes in each group. (F) DHI analysis. (G) MRI scoring analysis.

To investigate the role of SPP1 in disc degeneration, we performed imaging evaluations and protein expression analysis across different experimental groups. WB results (Fig. 8A–C) showed a significant increase in the expression of SPP1 and CD44 in the surgical group, indicating that SPP1 plays a key role in disc degeneration. This upregulation of SPP1 and CD44 further supports the involvement of these proteins in the progression of disc degeneration.

Imaging evaluations (Fig. 8D) demonstrated structural changes in the discs across the different experimental groups. Notably, the SPP1 inhibition group showed less degeneration in both 2D and 3D imaging, particularly in the sagittal and coronal planes. These results suggest that inhibition of SPP1 helps maintain disc structure and reduces degeneration. X-ray and MRI images (Fig. 8E) further confirmed these findings, with a significant reduction in disc degeneration observed in the SPP1 inhibition group, highlighting its protective role in the intervertebral disc.

Quantitative analysis of disc height index (Fig. 8F) and MRI scoring (Fig. 8G) also showed improvements in disc height and a reduction in degeneration in the SPP1 inhibition group. These results suggest that SPP1 inhibition may slow the disc degeneration process, offering a potential therapeutic strategy for treating IVDD. Overall, the findings underscore the crucial role of SPP1 in the pathophysiology of disc degeneration and demonstrate that its inhibition can effectively mitigate degeneration, preserving disc integrity.

### MR analysis of SPP1 and CD44 effects on IVDD

**Figure 9.**
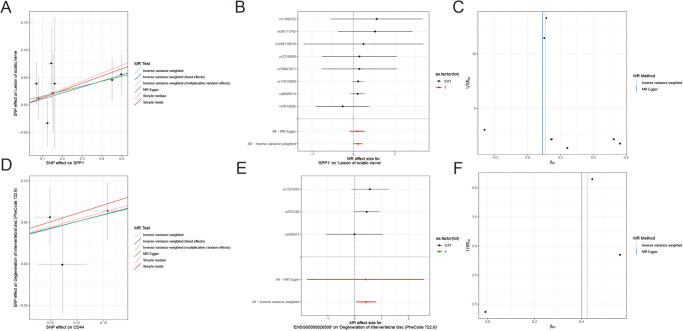
MR analysis of SPP1 and CD44 effects on IVDD. (A) MR analysis of SNP effects on SPP1 and their association with lesion of the sciatic nerve. (B) Forest plot illustrating MR effect size for SPP1 on sciatic nerve lesions. (C) Scatter plot of MR estimates for SPP1 with 1/S.E. vs βIV. (D) MR analysis of SNP effects on CD44 and their association with intervertebral disc degeneration. (E) Forest plot illustrating MR effect size for CD44 on intervertebral disc degeneration. (F) Scatter plot of MR estimates for CD44 with 1/S.E. vs βIV. Data show consistent associations between genetic variants of SPP1 and CD44 with intervertebral disc degeneration, with the strongest effects observed using the inverse variance weighted method.

MR was conducted to evaluate the causal relationship between SPP1, CD44, and IVDD. The MR analysis (Fig. 9A) revealed a significant positive association between SPP1 genetic variants and the degeneration of the sciatic nerve, indicating that SPP1 may play a role in nerve degeneration associated with disc degeneration. The SNP effects on SPP1 (Fig. 9A and D) demonstrated consistent positive associations across different MR methods, including IVW and MR Egger, suggesting robustness in the findings. Similarly, CD44 genetic variants showed a significant association with IVDD (Fig. 9D), with positive SNP effects on the degeneration of intervertebral disc structures. These findings suggest that both SPP1 and CD44 contribute to disc degeneration and may represent potential therapeutic targets for mitigating IVDD (Supplemental Digital Content Table 1, available at: http://links.lww.com/JS9/F266).

### Single-cell transcriptomic analysis of human nucleus pulposus tissues highlights the role of SPP1 + CD44 + Cells in intervertebral disc degeneration

scRNA-seq of NP tissue from patients with mild and severe IVDD revealed significant cellular and molecular changes. PCA maps (Supplemental Digital Content Fig. S11A, available at: http://links.lww.com/JS9/F265) showed distinct cell aggregates in mildly and severely degenerated tissues, and UMAP analysis (Supplemental Digital Content Fig. S11B, available at: http://links.lww.com/JS9/F265) further distinguished the two groups, reflecting transcriptional differences. SPP1 expression was elevated in severely degenerated tissues (Supplemental Digital Content Fig. S11C, available at: http://links.lww.com/JS9/F265), suggesting a role in matrix degradation and immune response, supported by the higher SPP1 and CD44 expression in severely degenerated tissues (Supplemental Digital Content Fig. S11D and F, available at: http://links.lww.com/JS9/F265). Cd44-expressing UMAP (Supplemental Digital Content Fig. S11E, available at: http://links.lww.com/JS9/F265) enhanced its involvement in immunomodulation and ECM remodeling. Dividing NP cells into four subpopulations (Supplemental Digital Content Fig. S11G, available at: http://links.lww.com/JS9/F265) showed a change in cell ratio between mild and severe degeneration, with an increase in double-positive cells in severe degeneration (Supplemental Digital Content Fig. S11H, available at: http://links.lww.com/JS9/F265). Heat-map analysis (Supplemental Digital Content Fig. S11I, available at: http://links.lww.com/JS9/F265) highlights ECM remodeling and immune pathways in severe degeneration. The stacked bars, Supplemental Digital Content Fig. S10J, available at: http://links.lww.com/JS9/F265, indicate a cell transition toward proinflammatory and matrix-degrading cells in the severely degenerated nucleus pulposus in humans, underlining the central role of SPP1 + CD44 + cells in IVDD progression.

## Discussion

In this study, we performed an in-depth investigation of the mechanisms by which notochord cells contribute to IVDD, utilizing an innovative mechanical pressure model, single-cell transcriptomics, and a detailed analysis of notochord cell function, revealing the pivotal role of notochord cells in the IVDD process^[43,44]^. To simulate the physiological environment of IVDD, we developed a rat mechanical pressure model. This model successfully replicates the IVDD microenvironment, enabling us to mimic the impact of mechanical stress on disc degeneration in rats, as experienced by humans^[45]^. By comparing the transcriptomic data of intervertebral discs under different pressure conditions with those of the control group, we found that rats under pressure conditions exhibited gene expression features associated with IVDD, including the upregulation of ECM degradation-related genes and the activation of immune response-related genes.

Through single-cell transcriptomic analysis, we identified distinct subpopulations of notochordal cells involved in IVDD. These notochordal cell populations exhibited significant differences in their gene expression profiles, reflecting their functional diversity in IVDD^[46,47]^. The inflammatory IMNC exhibited high levels of immune-related genes, indicating their potential role in immune regulation and modulation within the degenerating disc. On the other hand, the MFNCs were enriched in genes related to ECM synthesis, such as COL2A1, suggesting their involvement in maintaining disc integrity. Additionally, the SRNC showed increased expression of genes associated with stress response pathways, such as NF-kB, pointing to their role in responding to mechanical stress during degeneration. Our study also revealed that these notochordal cells interact closely with immune cells, particularly macrophages. Under mechanical stress, the interaction between IRNC and macrophages was enhanced, suggesting that these cells may influence immune responses through the secretion of cytokines and chemokines. This interaction highlights that notochordal cells not only contribute to the structural aspects of the disc but also play an active role in modulating the immune environment, which influences the progression of disc degeneration. These findings provide new insights into the multifaceted role of notochordal cells in IVDD, underscoring their involvement in both matrix remodeling and immune regulation.

Through the SPP1 and CD44-based classification approach, we were able to gain a more detailed understanding of the role of SPP1 in notochordal cells. Specifically, the SPP1 +/CD44 + and SPP1 +/CD44 − subpopulations represent changes in SPP1 expression under different conditions, which helps reveal its potential role in disc degeneration or other pathological states. By distinguishing these cell populations, we can gain deeper insights into how SPP1 interacts with immune responses, ECM remodeling, and cell migration, thus providing more precise cell-type-specific context and functional information regarding its key role in disc degeneration. This classification method offers a more detailed analytical perspective on the biological actions of SPP1. In our study, we found that SPP1 +/CD44 + notochordal cells play a crucial role in IVDD. These cells secrete SPP1, which is recognized by the CD44 receptor on macrophages within the disc, thereby activating immune-related signaling pathways and promoting macrophage polarization. This interaction highlights the significant role of SPP1 in modulating immune responses and inflammation during disc degeneration. Moreover, SPP1 +/CD44 − notochordal cells are involved in ECM synthesis and remodeling, contributing to the maintenance of disc structure and stability. This classification strategy provides a more nuanced analysis of SPP1’s biological role in IVDD by combining its interaction with immune responses, ECM remodeling, and cell migration^[21,48,49]^. The findings underscore the importance of SPP1 in immune modulation and matrix degradation, and suggest that its inhibition could be a potential therapeutic strategy for slowing down disc degeneration. MR analysis results also support a significant genetic association between SPP1 and CD44 and IVD, highlighting their potential causal role in the disease. These findings confirm the hypothesis that SPP1 and CD44 are involved in IVDD in human populations, suggesting that modulation of these targets could provide new therapeutic strategies for IVDD. These insights offer a more comprehensive understanding of the complex cellular mechanisms at play and support the potential of SPP1 as a therapeutic target in disc degeneration^[50–53]^.

Our findings extend and deepen previous studies, underscoring the complex dual role of notochord cells in both maintaining IVD homeostasis and contributing to disease progression^[22,54,55]^. By using an advanced rat disc degeneration pressure model, we created a more refined platform for studying the pathogenesis and potential treatment strategies for IVDD. Unlike prior research, our study employed cutting-edge single-cell transcriptomic analysis, which allowed us to uncover the significant heterogeneity and functional diversity within notochord cells^[56–59]^. This methodological approach offers a deeper understanding of how different notochordal subpopulations contribute to disc degeneration, highlighting their roles in immune modulation, ECM remodeling, and matrix degradation. By classifying notochordal cells based on gene expression profiles and functional characteristics, we identified distinct subpopulations with specific contributions to IVDD. This classification enhances our understanding of notochordal cell function and provides a clearer perspective on their interactions with immune cells.

However, our study has some limitations. First, it primarily relies on a rat IVDD model, and there are inherent differences between animal models and human physiology that may limit the direct extrapolation of results^[60]^. As pointed out, a more critical evaluation of the limitations inherent in the rat model is needed, particularly the interspecies differences in disc biology and immune responses, which could affect the relevance of our findings to human conditions. Although rat models are widely used in IVDD research, their similarity to human diseases can be limited in certain aspects, such as differences in disc structure and immune response pathways^[61,62]^. To improve the clinical relevance of these findings, future research should consider using larger animal models or conducting experiments in nonhuman primates, which provide a better representation of human disc degeneration and immune interactions^[63]^. Additionally, we explored the idea of directly injecting cells into animals as a potential treatment for IVDD. However, cell transplantation presents several challenges, including cell survival, directed migration, integration, and the risk of immune responses^[64–66]^. Specifically, xenogeneic transplantation could trigger immune rejection, which would require the use of immunosuppressive treatments. These challenges can complicate the evaluation of transplantation efficacy^[67,68]^. Therefore, future studies should focus on optimizing cell transplantation techniques, possibly using autologous cells or gene editing technologies to reduce immune rejection and improve the success of these treatments^[69]^.

## Conclusions

This study improves the mechanical compression model, better simulating the intervertebral disc’s mechanical environment and enhancing the clinical relevance of in vitro results. Single-cell transcriptomic analysis provided new insights into notochord cell functions, identifying potential targets for immune regulation and matrix metabolism. The study also explored the synergistic role of notochord cells in immune response and mechanical adaptation, with potential applications in developing new interventions, cell transplantation techniques, and drug screening. Future research should evaluate the long-term effects of notochord cells in larger animal models or clinical trials and explore enhancing their therapeutic efficacy using biomaterials or gene-editing approaches. Overall, this study offers valuable insights into the pathogenesis of IVDD and its immune-mechanical interactions, providing a foundation for clinical translational research.

## Data Availability

All data are available from the corresponding authors upon reasonable request.
